# What behaviour change technique content is offered to service users of the nationally implemented English NHS Digital Diabetes Prevention Programme: Analysis of multiple sources of intervention content

**DOI:** 10.1016/j.pmedr.2023.102112

**Published:** 2023-01-18

**Authors:** Rhiannon E. Hawkes, Lisa M. Miles, David P. French

**Affiliations:** Manchester Centre for Health Psychology, Division of Psychology and Mental Health, University of Manchester, UK

**Keywords:** Diabetes prevention, Digital interventions, Behaviour change, Fidelity, Implementation, Behaviour change techniques

## Abstract

The National Health Service Digital Diabetes Prevention Programme (NHS-DDPP) is a behavioural intervention for adults in England at risk of developing Type 2 diabetes, rolled out nationally via independent providers and their digital partners. The NHS England programme specification indicated 19 behaviour change techniques (BCTs) which should be present in the intervention, including BCTs to support self-regulation. A previous evaluation of the face-to-face service found an under-delivery of some self-regulatory BCTs. This study assessed whether those 19 BCTs were offered in the different interventions delivered by four digital providers.

A cross-sectional analysis of BCT content in the NHS-DDPP was elicited from the following sources: (a) online platforms (e.g. apps), (b) educational materials, and (c) health coaching (assessed via interviews with health coaches and audio-recorded telephone consultations). All materials were coded using the Behaviour Change Technique Taxonomy v1.

A total of 17, 15, 15 and 14 of the 19 specified BCTs were identified across the four digital provider programmes. A mean proportion of 43% of specified BCTs were present via providers’ apps, 74% via educational materials, and 62% via health coaching. An additional 41 BCTs were included in at least one of the four providers’ programmes.

Fidelity of BCT content in the NHS-DDPP was better than previously identified for the face-to-face DPP service. However, BCTs were offered via multiple modalities, thus the degree to which users engage with the modalities will impact on their exposure to BCTs and likely effectiveness of the digital programme.

## Highlights

• The NHS Digital Diabetes Prevention Programme has demonstrated good fidelity.
• There was variation in modes of delivery of intervention content across providers.
• All providers relied on educational materials to deliver intervention content.
• Some providers also relied on health coach support to deliver intervention content.
• Research is needed to assess engagement with these different modalities.

## 1 Introduction

In response to the increasing incidence of Type 2 diabetes mellitus (T2DM) worldwide, diabetes prevention programmes have been implemented globally to target those individuals at risk of developing T2DM. Following international evidence, (Gong et al., 2019, Tuomilehto et al., 2001, Knowler et al., 2002, Kosaka et al., 2005, Ramachandran et al., 2006) the National Health Service (NHS) in England launched the NHS Diabetes Prevention Programme (NHS-DPP) in 2016 delivered by independent providers (NHS, 2022). Each provider offered a nine-month intervention to promote weight loss via increased physical activity and improved diet for adults at increased risk of developing T2DM. Initial results suggest that the NHS-DPP has worked in helping individuals to reduce their risk of progressing to T2DM (Valabhji et al., 2020, Marsden et al., 2022, McManus et al., 2022).

However, weaknesses with access and reach of the NHS-DPP (e.g. participation of younger adults) have been identified (Howarth et al., 2020). To address this, in 2019 a digital pathway was offered as an alternative to the face-to-face programme. Four independent digital providers were commissioned to deliver the NHS Digital Diabetes Prevention Programme (NHS-DDPP). They were required to follow a programme specification detailing the key intervention features of the programme (NHS England, 2019), based on the current evidence (Ashra et al., 2015, National Institute for Health and Care Excellence, 2017). The NHS-DPP programme specification indicated 19 behaviour change techniques (BCTs) that should be present within the intervention (NHS, 2019, National Institute for Health and Care Excellence, 2017). BCTs are defined as the ‘active ingredients’ of an intervention to promote behaviour change (e.g. setting goals, providing support) (Michie et al., 2013). The programme specification particularly emphasised the use of BCTs to self-regulate behaviours (e.g. goal setting, self-monitoring), as evidence suggests these BCTs are important for achieving behavioural change in diet and physical activity behaviours (National Institute for Health and Care Excellence, 2017).

It is currently unclear whether the digital NHS-DDPP providers are delivering those 19 BCTs with fidelity to the specification. Assessing fidelity is important because: (1) the BCTs described in the programme specification are the components most supported by evidence of effectiveness in changing health behaviours; (2) given that there are four independent providers delivering the digital programme, there may be unwarranted variation in delivery of BCTs; and (3) if NHS-DDPP providers are not delivering the 19 BCTs specified in the programme specification, and instead delivering different BCTs, reasons for (in)effectiveness cannot be established (Bellg et al., 2004).

Previous research on the face-to-face NHS-DPP has produced a thorough assessment of fidelity (Hawkes et al., 2022). This previous programme of work identified the 19 BCTs included in the NHS-DPP programme specification (Hawkes et al., 2020). Analysis of delivery in the face-to-face programme found that although providers were good at delivering the BCTs in their programme manuals, there was an under-delivery of some key 19 BCTs, notably those which focused on self-regulation of behaviours such as problem solving and reviewing goals (French et al., 2021).

The present research evaluates the digital version of the programme and extends the previous analysis by also considering the *mode of delivery* of BCTs in the four providers’ digital programmes. It is increasingly recognised that the mode of delivery of intervention techniques (i.e. *how* a BCT is delivered) can impact on their effectiveness (Dombrowski et al., 2016, Marques et al., 2020, Black et al., 2020 Sep). Because service users can use the digital programmes to suit their own needs (e.g. choosing one mode of delivery over another), the present study looks at what is ‘offered’ across each of the four programmes. For example, service users may not engage in support or information about reducing alcohol intake if they do not consume alcohol, but this may be *offered* as part of that provider’s digital programme. Thus, the current study analyses all materials made available to the research team that were part of each provider’s digital programmes, whether or not service users chose to take them up.

The overall aim of the present research was to examine fidelity of the NHS-DDPP offer to programme specifications. The current fidelity analysis relates specifically to the intervention content in the NHS-DDPP (i.e. the BCTs), rather than the broader aspects of fidelity such as the extent to which an intervention is put into practice as intended (Carroll et al., 2007). Specific objectives were: (a) to describe which BCTs were offered across each of the four digital provider interventions, including variation across provider programmes; (b) to compare whether the 19 BCTs in the programme specification were actually present in each of the four providers’ digital interventions; and (c) to assess whether there was variation in the mode of delivery of BCTs offered across each of the four provider interventions.

## 2 Methods

### 2.1 Design

We conducted a cross-sectional analysis of BCT content for the four digital provider programmes. BCT content was collected via multiple modalities made available to the research team, to allow researchers to obtain the most comprehensive understanding of all the content of each programme. BCTs offered across the different modalities for the four providers were compared with the NHS-DPP programme specification (Hawkes et al., 2020). The authors checked that these specification documents had not been updated and there were no further specifications to consider for the digital programme.

### 2.2 Sample

The four digital providers were private service organisations who each secured contracts to deliver the NHS-DDPP in 2019–2021. Three of the four digital providers (Oviva, Second Nature and Liva) were in partnership with one of the face-to-face providers of the NHS-DPP to deliver the digital programme. One of the providers (WW) delivered both their face-to-face and digital pathways of the NHS-DPP. Providers are labelled A-D for the current manuscript to preserve anonymity.

### 2.3 Materials

BCT content was analysed across the following modalities for each of the four digital providers:

• Guest access to smartphone and web applications for three out of the four providers (which included tracking technology, articles, videos, recipes, and group support forums). The other provider supplied an app user guide.
• All educational materials, including learning platforms (containing articles, videos, podcasts and quizzes), online articles, PDF articles, videos, online workbooks and additional workbooks posted to service users.
• Standard text/script sent to service users via email and text messages (e.g. reminders to monitor weight, re-engagement messages).
• Transcripts of semi-structured qualitative interviews with health coaches who were involved in the delivery of the digital programme across the four providers.
• Transcripts of one-to-one telephone consultations between health coaches and service users for Provider A.

### 2.4 Procedures

This work was reviewed and approved by the North West Greater Manchester East NHS Research Ethics Committee (Reference: 17/NW/0426, 1st August 2017). Authors were in contact with the management staff of each of the four NHS-DDPP providers to obtain all relevant documentation and gain access to all online platforms containing content for each digital programme.

#### 2.4.1 Interview recruitment

NHS-DDPP health coaches employed by each of the four digital providers were involved in delivering the digital programme content to service users. They were recruited to the study by email via digital provider leads. Health coaches who were interested in taking part contacted the research team to arrange an interview via a video call platform (Zoom) with one of two researchers (REH and LMM) and interviews were transcribed verbatim. Full recorded verbal consent was obtained from each participant prior to commencing the interview.

The interviews were semi-structured; researchers initially asked open questions about general topics, followed by more detailed probing on specific issues. Interviews covered the following topics:

• Participants’ professional background.
• Participants’ role in delivering the behaviour change programme content.
• Participants’ role in supporting service users throughout the programme (e.g. at first contact, continued engagement, moderating support forums).
• Content of the digital intervention, and the format of intervention features included in the programme.

We also obtained a sample of audio-recorded health coach consultations to provide a more comprehensive understanding of the health coach support delivered on Provider A’s programme as one-to-one telephone support calls was part of their service provision. Following an email from the health coach leads, those health coaches interested in taking part contacted the research team for more information. Full recorded verbal consent was obtained from health coaches, prior to the research team receiving data. Service users who consented to having their audio-recorded calls used for research purposes were initially told about the research when first enrolled onto the programme and emailed a participant information sheet. The health coach took full recorded verbal consent with the service user during their first telephone consultation. Audio files were sent securely to the research team, anonymised, and transcribed verbatim.

### 2.5 Data analysis

#### 2.5.1 Coding framework and procedures

All documents, online platforms, interview transcripts and telephone consultation transcripts were coded separately using the Behaviour Change Technique Taxonomy (BCTTv1) (Michie et al., 2013). BCTs were coded using an author-developed data extraction sheet. Researchers underwent training in the use of the BCTTv1 (BCTTv1 online training, 2022). When coding the health coach telephone consultations for provider A, only BCTs delivered by the health coach were coded (i.e. not those mentioned by the service user). A set of coding rules were developed through team discussions, following guidance from taxonomy authors. See Appendix A for further details on BCT coding rules developed and Appendix B for BCT definitions.

Ten percent of delivery documentation from each of the four providers were double coded. Interrater reliability (IRR) was calculated using Cohen’s Kappa (Landis and Koch, 1977) to determine consistency between coders. Identified coding discrepancies were discussed between three authors until consensus was agreed.

#### 2.5.2 Fidelity analysis

The BCTs present in all modalities of delivery for each provider were compared with the 19 BCTs specified in the programme specification (NHS, 2019, National Institute for Health and Care Excellence, 2017) and results were tabulated. Sensitivity analyses were conducted to establish whether results were affected by including further BCTs that were either (a) included as external sources only (e.g. users signposted to external webpages), (b) prompted to users rather than explicitly delivered via the programme materials (e.g. prompting users to book an appointment with their GP to receive feedback on their HbA1c [‘Biofeedback’]), (c) mentioned in delivery documentation that service users would receive something but there was no direct evidence of BCT delivery (e.g. social support from health coach), or (d) present in optional programme materials (e.g. optional extra articles).

## 3 Results

Table 1 details the programme delivery materials obtained from each provider. A total of two, four, four and two interviews (n = 12) were conducted with health coaches from providers A, B, C and D respectively. Provider A shared a sample of audio-recorded telephone consultations between health coaches and service users (n = 2 service users at four and two time-points respectively, n = 6 calls). See Table 2 detailing the characteristics of health coaches included in the present study.

**Table 1:** Programme delivery materials obtained from each of the four digital providers.

	**Provider A**	**Provider B**	**Provider C**	**Provider D**
**Delivery materials analysed**				
**Content included**				
Access to app / online educational content	√	√	√	√
Educational leaflets / workbooks	n/a	√	√	√
**How content was assessed**				
Access to app / online educational content	42 online modules; App user guide obtained	App access; Weekly online articles	App access	App access; Online articles unlocked daily over 12 weeks, plus 8 optional 4-week courses
Educational leaflets / workbooks	n/a (all educational content was accessed via online modules)	NDPP handbook	PDF documents, videos and links sent to service users	Nutritional handbook and recipe book
**Transcripts analysed**				
No. of interviews with health coaches employed by each provider	2	4	4	2
Audio recordings of health coach consultations with service users a	√(n = 2 service users at two and four time points, n = 6)	n/a	x(not routinely recorded)	n/a

*Note.* n/a indicates that provider did not deliver content via that modality.

a Regular phone calls were not part of the service provision for providers B and D, and provider C was unable to provide data on the support between health coaches and services users as these calls were not routinely recorded.

**Table 2:** Characteristics of health coaches who took part in telephone interviews and one-to-one telephone consultations.

	**Provider A**	**Provider B**	**Provider C**	**Provider D**
**Health coach interviews**				
No. of health coaches interviewed	2	4	4	2
Length of time working for provider (range)	1 – 2 years	2 – 10 years	3 months – 1.5 years	1.5 – 2 years
Length of time delivering NHS-DDPP (range)	12 – 14 months	9 months – 24 months	3 months – 24 months	9 – 12 months
**One-to-one telephone consultations**				
No. of health coaches recruited	2	–	–	–

*Note.* The health coaches who provided audio-recorded telephone consultations for provider A were a different sample to those health coaches who were interviewed for this provider.

### 3.1 BCTs present across all modalities of the four digital programmes

Average kappa values across providers ranged from 0.64 to 0.75 for the double-coding of BCTs in providers’ delivery documentation, demonstrating moderate agreement between coders (McHugh, 2012), prior to resolving discrepancies (see Table A2 of Appendix C for all kappa values).

A total of 47, 44, 41 and 46 unique BCTs were identified across all modes of delivery within provider A, B, C and D’s digital programmes respectively. Self-regulatory BCTs were identified for all digital providers. In addition, providers A, B and D included a further four, two and four unique BCTs respectively within their programmes, which were either signposted via external webpages only, prompted to users rather than explicitly delivered via the programme materials, or present in optional programme material. These further BCTs have been included in Table A3 of Appendix D for sensitivity analyses.

### 3.2 Fidelity analysis: BCTs present in the full programme specification compared to BCTs present in providers’ digital interventions

Of the 19 BCTs in the NHS-DPP programme specification, a total of 17 (89 %), 15 (79 %), 15 (79 %) and 14 (74 %) BCTs were present across provider A-D’s digital programmes respectively, giving an overall mean proportion of BCTs present to the programme specification of 80 % (see Table 3). A total of 30, 29, 26 and 32 non-specified BCTs were identified across provider A-D’s digital programmes respectively that were not in the programme specification, 16 of which were common across all programmes, including: commitment, feedback on outcomes of behaviour, information about emotional consequences, information about social and environmental consequences, prompts/cues and self-reward (see Table A3 of Appendix D).

**Table 3:** Fidelity of behaviour change technique intervention content to the full programme specification.

**Behaviour Change Techniques**	**Programme specification**	**Provider A**	**Provider B**	**Provider C**	**Provider D**^a^
Goal setting (behaviour)	√	√	√	√	√
Goal setting (outcome)	√	√	√	√	√
Graded tasks	√	√	√	√	√
Information about health consequences	√	√	√	√	√
Social support (unspecified)	√	√	√	√	√
Action planning	√	√	√	√	√
Behaviour substitution	√	√	√	√	√
Feedback on behaviour	√	√	√	√	√
Problem solving	√	√	√	√	√
Self-monitoring of behaviour	√	√	√	√	√
Self-monitoring of outcome(s) of behaviour	√	√	√	√	√
Behavioural practice/rehearsal	√	√	√	√	√
Review outcome goal(s)	√	√	√	√	
Credible source	√	√		√	√
Social support (practical) b	√	√	√		
Pros and cons	√	√		√	
Social support (emotional) b	√		√		√
Pharmacological support	√	√			
Monitoring of outcome(s) of behaviour without feedback	√				
**PERCENTAGE agreement (BCTs delivered in line with programme specification)**		**89 %**	**79 %**	**79 %**	**74 %**

*Note.* Delivery of BCTs in the above table include data from all intervention materials (for all providers), health coach interviews (for all providers) and analysis of one-to-one calls between health coach and service user (for Provider A only).

a We were not able to analyse the group support functionalities offered by all providers due to data protection considerations. Results may have differed if the research team could access these groups. This may impact results for Provider D who delivered health coaching via moderation of peer support groups.

b ‘Social support (practical)’ and ‘Social support (emotional)’ were coded as one behaviour change technique in the programme specification, as it stated that either of these forms of social support could be delivered.

### 3.3 Variation in BCTs offered via different modalities across each of the four digital interventions

Table 4 shows a breakdown of the modes of delivery of the 19 specified BCTs across each of the four providers. A mean proportion of 43 % of those 19 specified BCTs were present via apps, 74 % via educational materials, and 62 % via health coach support. Providers A, B and D delivered the highest proportion of these BCTs via educational materials (84 %, 74 % and 74 % respectively), whereas provider C delivered the highest proportion of these specified BCTs via support from health coaches (68 %). Provider A also reported to deliver 74 % of those specified BCTs via health coach support.

**Table 4:** Mode of delivery of the 19 specified behaviour change techniques that were present in providers’ digital programmes.

	**Provider A**			**Provider B**			**Provider C**			**Provider D**^a^		
**Behaviour Change Techniques**	**App**^b^	**Education materials**	**Health coach**^c^	**App**	**Education materials**	**Health coach**^c^	**App**	**Education materials**	**Health coach**^c^	**App**	**Education materials**	**Health coach**^c^
Social support (unspecified)	√	√	√	√	√	√	√	√	√	√	√	√
Self-monitoring of outcome(s) of behaviour	√	√	√	√	√		√	√	√	√	√	√
Self-monitoring of behaviour	√	√	√	√	√		√		√	√	√	√
Action planning	√	√	√		√	√	√	√	√	√	√	√
Problem solving	√	√	√		√	√		√	√	√	√	√
Social support (practical)	√	√			√							
Feedback on behaviour	√	√	√	√			√		√	√	√	√
Goal setting (behaviour)	√	√	√		√	√	√	√	√	√	√	√
Goal setting (outcome)	√	√	√		√	√	√	√	√	√	√	√
Behaviour substitution		√	√		√	√		√	√	√	√	
Information about health consequences		√	√		√	√		√	√	√	√	√
Review outcome goal(s)		√	√		√			√				
Behavioural practice/rehearsal		√		√	√			√	√	√	√	√
Credible source		√	√					√	√	√	√	√
Graded tasks		√	√		√	√			√		√	√
Pharmacological support		√										
Pros and cons			√					√				
Social support (emotional)					√						√	
Monitoring of outcome(s) of behaviour without feedback												
**Totals:**	**9/19**	**16/19**	**14/19**	**5/19**	**14/19**	**8/19**	**7/19**	**12/19**	**13/19**	**12/19**	**14/19**	**12/19**
**Percent:**	**47 %**	**84 %**	**74 %**	**26 %**	**74 %**	**42 %**	**37 %**	**63 %**	**68 %**	**63 %**	**74 %**	**63 %**

a We were not able to analyse the group support functionalities offered by all providers due to data protection considerations. Results may have differed if the research team could access these groups. This may impact results for Provider D who delivered health coaching via moderation of peer support groups.

b Note that provider A only supplied research team with an app user guide, which outlined what was available via the app and how to use functions of the app, but researchers did not have direct access to the provider app. Thus, results may have differed if actual access to the app was provided.

c Data from interviews with health coaches about their job role as health coach on the NHS-DDPP, and includes data from audio-recorded telephone consultations between health coaches and service users for provider A.

Table A4 in Appendix E presents a full list of BCTs in each digital programme and their modes of delivery. Table A5 in Appendix F presents analyses including the mode delivery of BCTs that were either (a) prompted, (b) present in external links outside of the providers’ platform, (c) mentioned without further evidence of delivery, or (d) included in optional educational materials. Sensitivity analyses showed no change in overall pattern of results when these additional BCTs were included.

## 4 Discussion

The present study has found good fidelity of the BCT intervention contents to the programme specification. There was variation across providers with regards to non-specified BCTs and modality of BCT delivery. Although all four providers delivered the majority of BCTs via educational materials, two providers also had an emphasis on health coach delivery of BCT content, especially those BCTs used to support self-regulation.

## 5 Strengths and limitations

All key educational materials were elicited by the research team, and all materials and transcripts were reliably coded using standardised methods. Taken together, the access to online platforms, documentation from each digital provider, interviews with health coaches and analysis of one-to-one telephone consultations provided the most comprehensive understanding of the behaviour change content offered by the four providers delivering the NHS-DDPP and captured the various modes of delivery. Examinations of intervention fidelity where the research team was independent of those who developed each of the interventions are rare (Borrelli, 2011), especially across multiple providers.

Despite the efforts taken to elicit all relevant materials across each of the four digital programmes, the research team were not able to obtain all modalities of data for all providers. Provider A was unable to provide app access, however, we obtained an app user guide with step-by-step instructions on the use of different functions and behaviour change content, including screenshots of the interface. Further, this provider still demonstrated the highest fidelity to the programme specification across the four providers.

The research team were unable to access support calls between the health coach and service users for provider C as calls were not routinely recorded, nor were we able to analyse the group support functionalities offered by all providers due to data protection considerations. Researchers handled this missing data by focusing interview questions with health coaches to meet these gaps (e.g. specifically asking about their role in moderating the group support forums and the types of behaviour change support they would routinely deliver). This gave us a valid representation of the health coach and group support offered in the NHS-DDPP that could not be formally observed by the research team. There is a risk that the analysis of health coach interviews may have led to health coaches over- or under-reporting BCTs due to the self-report nature of this method. However, this was the best available method for the research team to understand the behaviour change support delivered via this modality in line with data protection considerations for this study. Further, the majority of BCTs that health coaches self-reported to deliver were already captured across other modalities of delivery for that provider, thus the results did not impact on the overall pattern of fidelity results.

### 5.1 Comparison to prior work

Previous research from this programme of work evaluating fidelity of design of the NHS-DDPP found that digital providers planned to deliver 85 % of BCTs specified in the programme specification (Hawkes et al., 2022). Together with the current results, the NHS-DDPP design and programme offering demonstrate higher fidelity than what was previously found for the face-to-face NHS-DPP (Hawkes et al., 2020, French et al., 2021). An examination of delivery fidelity of the face-to-face NHS-DPP found that only seven of the 19 specified BCTs (37 %) were delivered at all eight sites observed by the research team (French et al., 2021). Thus, fidelity of intervention content in the digital programme is substantially improved at 80 %. This may be as a result of lessons that have been learned by NHS England since programme rollout in 2016 (Hawkes et al., 2022). Additionally, digital interventions may allow higher fidelity as intervention materials can be standardised whereas human delivery cannot.

Overall, there was a high reliance of delivery of BCTs via educational materials. Although some providers used health coaches to also deliver important self-regulatory BCTs, those providers that do not rely on health coach delivery risk end-users not understanding some BCTs (e.g. action planning and problem solving), as suggested in prior research (Miles et al., 2021, Miles et al., 2023). Previous research has found that the use of communicative functions to deliver BCTs via digital interventions (e.g. having access to a health coach) was effective in supporting behaviour change (Webb et al., 2010). More recent research on the NHS-DDPP pilot found that having access to peer support and a telephone service was associated with a significantly greater reduction in weight compared to not having these features (Ross et al., 2022).

The impact of additional non-specified BCTs on the effectiveness of the NHS-DDPP is yet to be established. Some of the non-specified techniques offered in digital providers’ programmes have at least some evidence of effectiveness in interventions with similar populations or target behaviours as the NHS-DDPP. For example, the techniques ‘demonstration of the behaviour’ and ‘instruction on how to perform a behaviour’ have been associated with reduced blood glucose levels in people with T2DM (Cradock et al., 2017). It is possible that interventions containing more strategies to help people change their diet and physical activity behaviours may be more effective (Samdal et al., 2017, Van Rhoon et al., 2020), but there is a lack of evidence on how using more or less BCTs in diabetes prevention interventions can impact on outcomes.

### 5.2 Implications for practice

The present research has found that the NHS-DDPP shows good fidelity to the programme specification, demonstrating the benefit of digital interventions to deliver large-scale behaviour change support with higher fidelity. However, the emphasis of delivering much of this behaviour change intervention content via educational materials may not necessarily elicit engagement from service users as this is a passive means of delivery. For example, if service users do not engage with educational content they would receive a much lower percentage of BCTs. However, it is also likely in practice that these modes of delivery will be used in conjunction with one another, for example, the educational materials may be used alongside health coaching and the app throughout the programme.

This research has established substantial variation by digital providers in terms of modality of BCT delivery. Evaluations of the NHS-DDPP should consider this high heterogeneity when evaluating the digital programme so that conclusions can be drawn about what does and does not work in digital programmes.

### 5.3 Implications for research

Ongoing research will establish the extent to which service users engage with different features offered within the NHS-DDPP. For example, two digital providers relied more on health coach support during the programme, which may help promote user engagement with the programme (Yardley et al., 2016). Future research could also usefully examine the relative impacts of the four digital programmes on service user experience and behaviour change.

## 6 Conclusions

Fidelity of behaviour change content in the NHS-DDPP is promising, and better than that previously documented for the face-to-face programme. This suggests that high fidelity behaviour change content could be delivered at scale and at a lower cost. However, these BCTs are offered via multiple modalities, thus the degree to which users engage with the modalities will impact on their exposure to BCTs and likely effectiveness of the digital programme.

## 7 Ethics approval and consent to participate

The wider programme of research of which this study is a part of was reviewed and approved by the North West Greater Manchester East NHS Research Ethics Committee (Reference: 17/NW/0426, 1st August 2017). Full verbal consent was obtained from all participants included in this study.

## 8 Availability of data and materials

The materials from digital providers and audio-recordings of interviews analysed in the current study are not publicly available due to confidentiality agreements with the provider organisations, as some information is commercially sensitive. Some datasets are available from the corresponding author on reasonable request, although authors will require the explicit permission of the relevant provider organisations.

## Funding

This work is independent research funded by the National Institute for Health and Care Research (The Health and Social Care Delivery Research (HSDR) Programme, 16/48/07 – Evaluating the NHS Diabetes Prevention Programme (NHS DPP): the DIPLOMA research programme (Diabetes Prevention – Long Term Multimethod Assessment)). The views and opinions expressed in this manuscript are those of the authors and do not necessarily reflect those of the National Institute for Health and Care Research or the Department of Health and Social Care.

## CRediT authorship contribution statement

**Rhiannon E. Hawkes:** Methodology, Validation, Formal analysis, Data curation, Project administration, Investigation, Writing – original draft. **Lisa M. Miles:** Methodology, Investigation, Writing – review & editing, Project administration. **David P. French:** Funding acquisition, Conceptualization, Supervision, Writing – review & editing.

## Declaration of Competing Interest

The authors declare that they have no known competing financial interests or personal relationships that could have appeared to influence the work reported in this paper.

## Data Availability

Data will be made available on request.
