# Supplementary material for: What behaviour change technique content is offered to service users of the nationally implemented English NHS Digital Diabetes Prevention Programme: Analysis of multiple sources of intervention content
Source: Prev Med Rep. 2023 Jan 18;32:102112. doi: 10.1016/j.pmedr.2023.102112 (PMC9876742; doi:10.1016/j.pmedr.2023.102112)
Supplement: Supplementary data 1 [file mmc1.docx]

**Appendix A – BCT coding rules**

Data were extracted from the relevant documentation and interview transcripts using the BCTTv1 by one researcher (REH). Ten percent of each providers’ programme delivery documents were double coded for BCTs by a second researcher (LMM). Authors used a new author-developed data extraction sheet for each separate document and each individual interview for each provider.

Behaviour change technique coding procedures

BCTs were coded using an author-developed data extraction sheet. Researchers underwent training in the use of the BCTTv1 (<https://www.bct-taxonomy.com/>) and a set of coding rules were developed through team discussions. Behaviour change techniques (BCTs) were coded using an author-developed table, which included the following columns:

- BCT label
- Confidence of presence
- Information about behavioural targets (e.g. diet, physical activity)
- Mode of delivery (e.g. visual, auditory, text)
- Interactive vs. passive delivery of BCT (i.e. whether the planned BCT required action from the patients)
- Whether the planned BCT was optional or compulsory (e.g. an optional interactive activity)
- Location in the document/interview transcript (defined by page number and page quarter)
- Summarised evidence of the planned BCT

BCT coding rules stated that new BCTs would be coded on the commencement of a new activity or if a different health behaviour (e.g. diet, physical activity) was targeted. The level of target behaviour was also documented when coding the BCT ‘information about health consequences’ (e.g., levels of the target behaviour ‘diet’ included information about carbohydrates, fats, sugar, etc.) as the authors felt these were distinct pieces of information targeting distinct behaviours.

BCTs present in both the full programme specification and digital providers’ programmes were documented as indicating fidelity to the programme specification. BCTs stated in the full programme specification that were not present in providers’ programmes, and additional BCTs identified in the delivery documentation and interview transcripts which were not otherwise specified, were documented as indicating non-fidelity to the programme specification.

Sensitivity analyses

Where providers offered further BCTs within their programmes that were either (a) included as external sources only (e.g. users signposted to external webpages), (b) prompted to users rather than explicitly delivered via the programme materials (e.g. prompting users to book an appointment with their GP to receive feedback on their HbA1c [‘Biofeedback’], i.e. that BCT was not explicitly delivered via the provider), (c) mentioned in delivery documentation that service users *would* receive something but there was no direct evidence of this BCT within that document (e.g. social support from health coach), or (d) present in optional programme materials (e.g. optional extra articles), sensitivity analyses were conducted to assess whether these impacted on results.

**Appendix B – Behaviour change technique definitions**

**Table A1. Behaviour change technique definitions**

| **Behaviour Change Technique** | **Definition** |
| --- | --- |
| Goal setting for health behaviours [1.1] | Set or agree on a goal defined in terms of the behaviour to be achieved. |
| Problem solving [1.2] | Prompt the person to analyse factors influencing the behaviour and generate or select strategies that include overcoming barriers or increasing facilitators. |
| Goal setting for health outcomes [1.3] | Set or agree on a goal defined in terms of a positive outcome of wanted behaviour. |
| Action planning [1.4] | Prompt detailed planning of the performance of the behaviour (must include at least one of context, frequency, duration and intensity). |
| Reviewing behaviour goals [1.5] | Review behaviour goal(s) jointly with the person and consider modifying goal(s) or behaviour change strategy in light of achievement. |
| Discrepancy between current behaviour and goal [1.6] | Draw attention to discrepancies between a person’s current behaviour (in terms of the *form, frequency, duration, or intensity* of that behaviour) and the person’s previously set outcome goals, behavioural goals or action plans. |
| Reviewing outcome goals [1.7] | Review outcome goal(s) jointly with the person and consider modifying goal(s) in light of achievement. |
| Behavioural contract [1.8] | Create a written specification of the behaviour to be performed, agreed on by the person, and witnessed by another |
| Commitment [1.9] | Ask the person to affirm or reaffirm statements indicating commitment to change the behaviour. |
| Giving feedback on behaviour [2.2] | Monitor and provide informative or evaluative feedback on performance of the behaviour. |
| Self-monitoring of behaviour [2.3] | Establish a method for the person to monitor and record their behaviour(s) as part of a behaviour change strategy. |
| Self-monitoring of outcomes of behaviour [2.4] | Establish a method for the person to monitor and record the outcome(s) their behaviour as part of a behaviour change strategy. |
| Monitoring outcome of behaviour by others without feedback [2.5] | Observe or record outcomes of behaviour with the person’s knowledge as part of a behaviour change strategy. |
| Biofeedback [2.6] | Provide feedback about the body *(e.g. physiological or biochemical state)* using an external monitoring device as part of a behaviour change strategy. |
| Giving feedback on outcomes of behaviour [2.7] | Monitor and provide feedback on the outcome of performance of the behaviour. |
| Unspecified social support [3.1] | Advise on, arrange or provide social support or non-contingent praise or reward for performance of the behaviour. |
| Practical social support [3.2] | Advise on, arrange or provide practical help for performance of the behaviour. |
| Emotional social support [3.3] | Advise on, arrange or provide emotional social support for performance of the behaviour. |
| Instruction on how to perform a behaviour [4.1] | Advise or agree on how to perform the behaviour (includes ‘Skills training’). |
| Information about antecedents [4.2] | Provide information about antecedents (*e.g. social and environmental situations and events, emotions, cognitions)* that reliably predict performance of the behaviour. |
| Behavioural experiments [4.4] | Advise on how to identify and test hypotheses about the behaviour, its causes and consequences, by collecting and interpreting data. |
| Information about health consequences [5.1] | Provide information about health consequences of performing the behaviour. |
| Salience of consequences [5.2] | Use methods specifically designed to emphasise the consequences of performing the behaviour with the aim of making them more memorable (goes beyond informing about consequences). |
| Salience of behaviours ^a^ | Use methods specifically designed to emphasise the behaviour when linking the behaviour to the consequence with the aim of making the consequence more memorable. |
| Giving information about social and environmental consequences [5.3] | Provide information (e.g. written, verbal, visual) about social and environmental consequences of performing the behaviour. |
| Monitoring of emotional consequences [5.4] | Prompt assessment of feelings after attempts at performing the behaviour. |
| Anticipated regret [5.5] | Induce or raise awareness of expectations of future regret about performance of the unwanted behaviour. |
| Giving information about emotional consequences [5.6] | Provide information (e.g. written, verbal, visual) about emotional consequences of performing the behaviour. |
| Giving a demonstration of the behaviour [6.1] | Provide an observable sample of the performance of the behaviour, directly in person or indirectly e.g. via film, pictures, for the person to aspire to or imitate. |
| Social comparison [6.2] | Draw attention to others’ performance to allow comparison with the person’s own performance. |
| Prompts/cues [7.1] | Introduce or define environmental or social stimulus with the purpose of prompting or cueing the behaviour. |
| Remove access to the reward [7.4] | Advise or arrange for the person to be separated from situations in which unwanted behaviour can be rewarded in order to reduce the behaviour. |
| Remove aversive stimulus [7.5] | Advise or arrange for the removal of an aversive stimulus to facilitate behaviour change. |
| Behavioural practice [8.1] | Prompt practice or rehearsal of the performance of the behaviour in order to increase habit or skill. |
| Behaviour substitution [8.2] | Prompt the substitution of the unwanted behaviour with a wanted or neutral behaviour. |
| Habit formation [8.3] | Prompt rehearsal and repetition of the behaviour in the same context repeatedly so that the context elicits the behaviour. |
| Habit reversal [8.4] | Prompt rehearsal and repetition of an alternative behaviour to replace an unwanted habitual behaviour. |
| Overcorrection [8.5] | Ask to repeat the wanted behaviour in an exaggerated way following an unwanted behaviour. |
| Graded tasks [8.7] | Set easy-to-perform tasks, making them increasingly difficult, but achievable, until behaviour is performed. |
| Credible source [9.1] | Present verbal or visual communication from a credible source in favour of or against the behaviour. |
| Pros and cons [9.2] | Advise the person to identify and compare reasons for wanting (pros) and not wanting to (cons) change the behaviour. |
| Comparative imagining of future outcomes [9.3] | Prompt or advise the imagining and comparing of future outcomes of changed versus unchanged behaviour. |
| Material incentive (behaviour) [10.1] | Inform that money, vouchers or other valued objects will be delivered if and only if there has been effort and/or progress in performing the behaviour. |
| Material reward (behaviour) [10.2] | Arrange for the delivery of money, vouchers or other valued objects if and only if there has been effort and/or progress in performing the behaviour. |
| Social reward [10.4] | Arrange verbal or non-verbal reward if and only if there has been effort and/or progress in performing the behaviour. |
| Social incentive [10.5] | Inform that a verbal or non-verbal reward will be delivered if and only if there has been effort and/or progress in performing the behaviour. |
| Non-specific incentive [10.6] | Inform that a reward will be delivered if and only if there has been effort and/or progress in performing the behaviour. |
| Self-incentive [10.7] | Plan to reward self in future if and only if there has been effort and/or progress in performing the behaviour. |
| Self-reward [10.9] | Prompt self-praise or self-reward if and only if there has been effort and/or progress in performing the behaviour. |
| Reward (outcome) [10.10] | Arrange for the delivery of a reward if and only if there has been effort and/or progress in achieving the behavioural outcome. |
| Pharmacological support [11.1] | Provide, or encourage the use of or adherence to, drugs to facilitate behaviour change. |
| Reduce negative emotions [11.2] | Advise on ways of reducing negative emotions to facilitate performance of the behaviour. |
| Increase positive emotions ^b^ | Advise on ways of increasing positive emotions to facilitate performance of the behaviour. |
| Restructuring the physical environment [12.1] | Change, or advise to change the physical environment in order to facilitate performance of the wanted behaviour or create barriers to the unwanted behaviour. |
| Restructuring the social environment [12.2] | Change, or advise to change the social environment in order to facilitate performance of the wanted behaviour or create barriers to the unwanted behaviour. |
| Avoiding/reducing exposure to cues for the behaviour [12.3] | Advise on how to avoid exposure to specific social and contextual/physical cues for the behaviour, including changing daily or weekly routines. |
| Distraction [12.4] | Advise or arrange to use an alternative focus for attention to avoid triggers for unwanted behaviour. |
| Adding objects to the environment [12.5] | Add objects to the environment in order to facilitate performance of the behaviour. |
| Body changes [12.6] | Alter body structure, functioning or support directly to facilitate behaviour change. |
| Identification of self as role model [13.1] | Inform that one's own behaviour may be an example to others. |
| Framing/reframing [13.2] | Suggest the deliberate adoption of a perspective or new perspective on behaviour (e.g. its purpose) in order to change cognitions or emotions about performing the behaviour. |
| Reward alternative behaviour [14.8] | Arrange reward for performance of an alternative to the unwanted behaviour. |
| Verbal persuasion about capability [15.1] | Tell the person that they can successfully perform the wanted behaviour, arguing against self-doubts and asserting that they can and will succeed. |
| Mental rehearsal of successful performance [15.2] | Advise to practise imagining performing the behaviour successfully in relevant contexts. |
| Focus on past success [15.3] | Advise to think about or list previous successes in performing the behaviour (or parts of it). |
| Self-talk [15.4] | Prompt positive self-talk (aloud or silently) before and during the behaviour. |
| Vicarious consequences [16.3] | Prompt observation of the consequences (including rewards and punishments) for others when they perform the behaviour. |

*Note:* Definitions are summarised from BCTTv1. Numbers in square brackets are corresponding number in BCTTv1.

*^a^* Salience of behaviours was not listed in the BCTTv1, but has been identified as a new behaviour change technique by the authors of this paper.

*^b^* Increase positive emotions is not listed in the BCTTv1, but was noted by the authors for inclusion in the next version of the taxonomy.

**Appendix C – Kappa values**

**Table A2. Kappa values for NHS-DDPP documents**

| **Source document** | **Kappa value** |
| --- | --- |
| **Programme Specification** |  |
| NHS Service Specification | 0.84 |
| NICE PH38 Guideline | 0.87 |
| **Programme Delivery** |  |
| Provider A | 0.75 |
| Provider B | 0.75 |
| Provider C | 0.64 |
| Provider D | 0.73 |

**Appendix D – Further BCTs identified in providers’ programmes which were not explicitly present in provider’s delivery materials**

Provider A had four further BCTs which were either only present in external sources which were either signposted by the provider (e.g. signposting to external websites for more information; these included ‘identification of self as role model’, ‘material reward (behaviour)’ and ‘remove access to the reward’) or were only prompted but not actually delivered in their digital programme (e.g. advising to go to the GP to have follow-up HbA1c test taken; ‘biofeedback’).

Provider B had two further BCTs which were only present within external links within the webpages of the provider’s website (i.e. users had to click on another link and read/engage with that additional link in order for that BCT to be ‘delivered’; these included ‘imaginary reward’ and ‘self-talk’).

Provider D included four further BCTs which were only present in their optional articles (i.e. service users could opt to receive additional reading during months 6-9 of the programme; these BCTs included ‘review outcome goal(s)’, ‘associative learning’, ‘habit reversal’ and ‘mental rehearsal of successful performance’).

See Table A3 below. BCTs with a red tick are those which were not explicitly present in provider’s core delivery materials, but would have been delivered to users if they engaged in additional material, or sought additional support (e.g. from GP to receive ‘biofeedback’).

**Table A3. SENSITIVITY ANALYSIS: Fidelity of delivery of behaviour change techniques (BCTs) specified in the full programme specification, including BCTs present in external sources, prompted BCTs, mentioned BCTs and optional BCTs**

| **Behaviour Change Techniques** | **Programme specification** | **Provider A** | **Provider B** | **Provider C** | **Provider D *^a^*** |
| --- | --- | --- | --- | --- | --- |
| Action planning | **✓** | **✓** | **✓** | **✓** | **✓** |
| Behaviour substitution | **✓** | **✓** | **✓** | **✓** | **✓** |
| Behavioural practice/rehearsal | **✓** | **✓** | **✓** | **✓** | **✓** |
| Credible source | **✓** | **✓** |  | **✓** | **✓** |
| Feedback on behaviour | **✓** | **✓** | **✓** | **✓** | **✓** |
| Goal setting (behaviour) | **✓** | **✓** | **✓** | **✓** | **✓** |
| Goal setting (outcome) | **✓** | **✓** | **✓** | **✓** | **✓** |
| Graded tasks | **✓** | **✓** | **✓** | **✓** | **✓** |
| Information about health consequences | **✓** | **✓** | **✓** | **✓** | **✓** |
| Monitoring of outcome(s) of behaviour without feedback | **✓** |  |  |  |  |
| Pharmacological support | **✓** | **✓** |  |  |  |
| Problem solving | **✓** | **✓** | **✓** | **✓** | **✓** |
| Pros and cons | **✓** | **✓** |  | **✓** |  |
| Review outcome goal(s) | **✓** | **✓** | **✓** | **✓** | **✓** |
| Self-monitoring of behaviour | **✓** | **✓** | **✓** | **✓** | **✓** |
| Self-monitoring of outcome(s) of behaviour | **✓** | **✓** | **✓** | **✓** | **✓** |
| Social support (emotional) *^b^* | **✓** |  | **✓** |  | **✓** |
| Social support (practical) *^b^* | **✓** | **✓** | **✓** |  |  |
| Social support (unspecified) | **✓** | **✓** | **✓** | **✓** | **✓** |
| Adding objects to the environment |  |  | **✓** | **✓** | **✓** |
| Anticipated regret |  | **✓** | **✓** |  | **✓** |
| Associative learning |  | **✓** | **✓** |  | **✓** |
| Avoidance/reducing exposure to cues for the behaviour |  | **✓** | **✓** | **✓** | **✓** |
| Biofeedback |  | **✓** |  |  |  |
| Body changes |  | **✓** |  |  | **✓** |
| Commitment |  | **✓** | **✓** | **✓** | **✓** |
| Comparative imaginings of future outcomes |  | **✓** |  | **✓** |  |
| Demonstration of behaviour |  | **✓** | **✓** |  | **✓** |
| Discrepancy between current behaviour and goal |  |  |  |  | **✓** |
| Distraction |  | **✓** |  |  | **✓** |
| Feedback on outcome(s) of behaviour |  | **✓** | **✓** | **✓** | **✓** |
| Focus on past success |  | **✓** | **✓** | **✓** | **✓** |
| Framing/reframing |  | **✓** | **✓** | **✓** | **✓** |
| Habit formation |  | **✓** | **✓** | **✓** | **✓** |
| Habit reversal |  |  | **✓** | **✓** | **✓** |
| Identification of self as role model |  | **✓** |  |  |  |
| Identity associated with changed behaviour |  |  |  |  | **✓** |
| Imaginary reward |  |  | **✓** |  |  |
| Increase positive emotions *^c^* |  | **✓** | **✓** | **✓** | **✓** |
| Information about antecedents |  | **✓** | **✓** | **✓** | **✓** |
| Information about emotional consequences |  | **✓** | **✓** | **✓** | **✓** |
| Information about social and environmental consequences |  | **✓** | **✓** | **✓** | **✓** |
| Instruction on how to perform the behaviour |  | **✓** | **✓** | **✓** | **✓** |
| Material incentive (behaviour) |  |  | **✓** |  |  |
| Material reward (behaviour) |  | **✓** | **✓** |  |  |
| Mental rehearsal of successful performance |  | **✓** | **✓** | **✓** | **✓** |
| Monitoring emotional consequences |  | **✓** |  | **✓** | **✓** |
| Prompts/cues |  | **✓** | **✓** | **✓** | **✓** |
| Re-attribution |  |  |  |  | **✓** |
| Reduce negative emotions |  | **✓** | **✓** | **✓** | **✓** |
| Remove access to the reward |  | **✓** |  | **✓** | **✓** |
| Restructuring the physical environment |  | **✓** | **✓** | **✓** | **✓** |
| Restructuring the social environment |  | **✓** |  | **✓** | **✓** |
| Review behaviour goal(s) |  | **✓** | **✓** | **✓** | **✓** |
| Reward (outcome) |  | **✓** | **✓** | **✓** | **✓***^e^* |
| Salience of behaviours *^d^* |  | **✓** |  | **✓** | **✓** |
| Salience of consequences |  |  |  |  | **✓** |
| Self-reward |  | **✓** | **✓** | **✓** | **✓** |
| Self-talk |  | **✓** | **✓** |  |  |
| Social comparison |  |  |  |  | **✓** |
| Social incentive |  |  | **✓** |  |  |
| Social reward |  | **✓** | **✓** | **✓** | **✓** *^e^* |
| Valued self-identity |  | **✓** | **✓** |  |  |
| Verbal persuasion about capability |  |  | **✓** |  |  |

***Note.* BCTs with a red tick are the additional BCTs identified and included in the sensitivity analysis.**

*Note.* BCTs above the bold line are those 19 core BCTs specified in the full programme specification underpinning the NHS-DPP.

*Note.* Delivery of BCTs in the above table include data from all intervention materials (for all providers), health coach interviews (for all providers) and analysis of one-to-one calls between health coach and service user (for Provider A only).

*^a^* We were not able to analyse the group support functionalities offered by all providers due to data protection considerations. Results may have differed if the research team could access these groups. This may impact results for Provider D who delivered health coaching via moderation of peer support groups.

*^b^* ‘Social support (practical)’ and ‘Social support (emotional)’ were coded as one behaviour change technique in the programme specification, as it stated that either of these forms of social support could be delivered.

*^c^* Increase positive emotions is not listed in the BCTTv1, but was noted by the authors for inclusion in the next version of the taxonomy and used in Hawkes et al. (2020).

*^d^* Salience of behaviours was not listed in the BCTTv1, but has been identified as a new behaviour change technique by the authors of this paper and used in Hawkes et al. (2020).

*^e^* ‘Social reward’ and ‘Reward (outcome)’ were coded as one behaviour change technique for this provider, as it did not state whether the reward was for the behaviour or outcome.

**Appendix E**

**Table A4. Mode of delivery of all behaviour change techniques present via providers’ digital programmes**

|  | **Provider A** | | | **Provider B** | | | **Provider C** | | | **Provider D *^a^*** | | |
| --- | --- | --- | --- | --- | --- | --- | --- | --- | --- | --- | --- | --- |
| **Behaviour Change Techniques** | **App *^b^*** | **Education materials** | **Health coach *^c^*** | **App** | **Education materials** | **Health coach *^c^*** | **App** | **Education materials** | **Health coach *^c^*** | **App** | **Education materials** | **Health coach *^c^*** |
| Action planning | **✓** | **✓** | **✓** |  | **✓** | **✓** | **✓** | **✓** | **✓** | **✓** | **✓** | **✓** |
| Behaviour substitution |  | **✓** | **✓** |  | **✓** | **✓** |  | **✓** | **✓** | **✓** | **✓** |  |
| Behavioural practice/rehearsal |  | **✓** |  | **✓** | **✓** |  |  | **✓** | **✓** | **✓** | **✓** | **✓** |
| Credible source |  | **✓** | **✓** |  |  |  |  | **✓** | **✓** | **✓** | **✓** | **✓** |
| Feedback on behaviour | **✓** | **✓** | **✓** | **✓** |  |  | **✓** |  | **✓** | **✓** | **✓** | **✓** |
| Goal setting (behaviour) | **✓** | **✓** | **✓** |  | **✓** | **✓** | **✓** | **✓** | **✓** | **✓** | **✓** | **✓** |
| Goal setting (outcome) | **✓** | **✓** | **✓** |  | **✓** | **✓** | **✓** | **✓** | **✓** | **✓** | **✓** | **✓** |
| Graded tasks |  | **✓** | **✓** |  | **✓** | **✓** |  |  | **✓** |  | **✓** | **✓** |
| Information about health consequences |  | **✓** | **✓** |  | **✓** | **✓** |  | **✓** | **✓** | **✓** | **✓** | **✓** |
| Monitoring of outcome(s) of behaviour without feedback |  |  |  |  |  |  |  |  |  |  |  |  |
| Pharmacological support |  | **✓** |  |  |  |  |  |  |  |  |  |  |
| Problem solving | **✓** | **✓** | **✓** |  | **✓** | **✓** |  | **✓** | **✓** | **✓** | **✓** | **✓** |
| Pros and cons |  |  | **✓** |  |  |  |  | **✓** |  |  |  |  |
| Review outcome goal(s) |  | **✓** | **✓** |  | **✓** |  |  | **✓** |  |  |  |  |
| Self-monitoring of behaviour | **✓** | **✓** | **✓** | **✓** | **✓** |  | **✓** |  | **✓** | **✓** | **✓** | **✓** |
| Self-monitoring of outcome(s) of behaviour | **✓** | **✓** | **✓** | **✓** | **✓** |  | **✓** | **✓** | **✓** | **✓** | **✓** | **✓** |
| Social support (emotional) |  |  |  |  | **✓** |  |  |  |  |  | **✓** |  |
| Social support (practical) | **✓** | **✓** |  |  | **✓** |  |  |  |  |  |  |  |
| Social support (unspecified) | **✓** | **✓** | **✓** | **✓** | **✓** | **✓** | **✓** | **✓** | **✓** | **✓** | **✓** | **✓** |
| Adding objects to the environment |  |  |  |  | **✓** |  |  | **✓** |  |  | **✓** |  |
| Anticipated regret |  | **✓** |  |  | **✓** |  |  |  |  |  | **✓** |  |
| Associative learning |  |  | **✓** |  | **✓** |  |  |  |  |  |  |  |
| Avoidance/reducing exposure to cues for the behaviour |  | **✓** | **✓** |  | **✓** |  |  | **✓** |  |  | **✓** |  |
| Biofeedback |  |  |  |  |  |  |  |  |  |  |  |  |
| Body changes |  | **✓** | **✓** |  |  |  |  |  |  |  | **✓** |  |
| Commitment |  | **✓** |  |  | **✓** |  | **✓** |  |  |  | **✓** |  |
| Comparative imaginings of future outcomes |  | **✓** | **✓** |  |  |  |  |  | **✓** |  |  |  |
| Demonstration of behaviour |  | **✓** |  | **✓** | **✓** |  |  |  |  | **✓** | **✓** |  |
| Discrepancy between current behaviour and goal |  |  |  |  |  |  |  |  |  | **✓** |  |  |
| Distraction |  | **✓** |  |  |  |  |  |  |  | **✓** | **✓** |  |
| Feedback on outcome(s) of behaviour | **✓** | **✓** | **✓** | **✓** | **✓** |  | **✓** | **✓** |  | **✓** | **✓** |  |
| Focus on past success |  | **✓** | **✓** |  | **✓** |  |  | **✓** |  | **✓** | **✓** | **✓** |
| Framing/reframing |  | **✓** | **✓** |  | **✓** |  |  | **✓** |  | **✓** | **✓** | **✓** |
| Habit formation |  | **✓** |  |  | **✓** |  |  |  | **✓** |  | **✓** | **✓** |
| Habit reversal |  |  |  |  | **✓** |  |  | **✓** |  |  |  |  |
| Identification of self as role model |  |  |  |  |  |  |  |  |  |  |  |  |
| Identity associated with changed behaviour |  |  |  |  |  |  |  |  |  |  | **✓** |  |
| Imaginary reward |  |  |  |  |  |  |  |  |  |  |  |  |
| Increase positive emotions |  | **✓** |  |  | **✓** |  |  | **✓** |  | **✓** | **✓** |  |
| Information about antecedents |  | **✓** | **✓** |  | **✓** |  |  | **✓** |  | **✓** | **✓** | **✓** |
| Information about emotional consequences |  | **✓** | **✓** |  | **✓** |  |  | **✓** |  | **✓** | **✓** |  |
| Information about social and environmental consequences |  | **✓** |  |  | **✓** |  |  | **✓** |  |  | **✓** |  |
| Instruction on how to perform the behaviour |  | **✓** |  | **✓** | **✓** |  |  | **✓** |  | **✓** | **✓** |  |
| Material incentive (behaviour) |  |  |  |  | **✓** |  |  |  |  |  |  |  |
| Material reward (behaviour) |  |  |  |  | **✓** |  |  |  |  |  |  |  |
| Mental rehearsal of successful performance |  | **✓** |  |  | **✓** |  |  |  | **✓** |  | **✓** |  |
| Monitoring emotional consequences |  | **✓** | **✓** |  |  |  |  |  | **✓** |  |  |  |
| Prompts/cues |  | **✓** | **✓** |  | **✓** | **✓** | **✓** | **✓** | **✓** | **✓** | **✓** |  |
| Re-attribution |  |  |  |  |  |  |  |  |  | **✓** | **✓** | **✓** |
| Reduce negative emotions |  | **✓** | **✓** | **✓** | **✓** |  |  | **✓** |  | **✓** | **✓** |  |
| Remove access to the reward |  |  |  |  |  |  |  | **✓** |  |  | **✓** |  |
| Restructuring the physical environment |  | **✓** | **✓** |  | **✓** |  |  | **✓** | **✓** |  | **✓** |  |
| Restructuring the social environment |  |  | **✓** |  |  |  |  | **✓** | **✓** |  | **✓** |  |
| Review behaviour goal(s) | **✓** | **✓** | **✓** |  | **✓** |  |  | **✓** | **✓** |  | **✓** |  |
| Reward (outcome) |  | **✓** | **✓** |  | **✓** | **✓** |  |  | **✓** |  | **✓** |  |
| Salience of behaviours |  | **✓** |  |  |  |  |  | **✓** |  |  | **✓** |  |
| Salience of consequences |  |  |  |  |  |  |  |  |  |  | **✓** |  |
| Self-reward |  | **✓** |  |  | **✓** |  | **✓** |  |  |  | **✓** |  |
| Self-talk |  | **✓** |  |  |  |  |  |  |  |  |  |  |
| Social comparison |  |  |  |  |  |  |  |  |  | **✓** | **✓** | **✓** |
| Social incentive |  |  |  | **✓** | **✓** |  |  |  |  |  |  |  |
| Social reward |  | **✓** | **✓** | **✓** | **✓** | **✓** |  |  | **✓** |  | **✓** | **✓** |
| Valued self-identity |  | **✓** |  |  | **✓** |  |  |  |  |  |  |  |
| Verbal persuasion about capability |  |  |  |  | **✓** |  |  |  |  |  |  |  |
| **Totals:** | **11/47** | **44/47** | **31/47** | **11/44** | **43/44** | **11/44** | **11/41** | **30/41** | **23/41** | **26/46** | **45/46** | **19/46** |
| **Percent:** | **23%** | **94%** | **66%** | **25%** | **98%** | **25%** | **27%** | **73%** | **56%** | **57%** | **98%** | **41%** |

*Note.* BCTs above the bold line are those 19 BCTs which were specified for inclusion in the NHS-DPP programme specification.

*^a^* We were not able to analyse the group support functionalities offered by all providers due to data protection considerations. Results may have differed if the research team could access these groups. This may impact results for Provider D who delivered health coaching via moderation of peer support groups.

*^b^* Note that provider A only supplied research team with an app user guide, which outlined what was available via the app and how to use functions of the app, but researchers did not have direct access to the provider app. Thus, results may have differed if actual access to the app was provided.

*^c^* Data from interviews with health coaches about their job role as health coach on the NHS-DDPP, and includes data from audio-recorded telephone consultations between health coaches and service users for provider A.

**Appendix F – Mode of BCT delivery including BCTs which were either prompted (i.e. not directly delivered), present in external sources (i.e. not directly in provider’s digital DPP platforms), or present in optional materials**

**Table A5. SENSITIVITY ANALYSIS: Mode of delivery of BCTs present via providers’ digital programmes, including BCTs which were either prompted, present in external sources, or present in optional materials**

|  | **Provider A** | | | **Provider B** | | | **Provider C** | | | **Provider D *^a^*** | | |
| --- | --- | --- | --- | --- | --- | --- | --- | --- | --- | --- | --- | --- |
| **Behaviour Change Techniques** | **App *^b^*** | **Education materials** | **Health coach *^c^*** | **App** | **Education materials** | **Health coach *^c^*** | **App** | **Education materials** | **Health coach *^c^*** | **App** | **Education materials** | **Health coach *^c^*** |
| Action planning | **✓** | **✓** | **✓** |  | **✓** | **✓** | **✓** | **✓** | **✓** | **✓** | **✓** | **✓** |
| Behaviour substitution |  | **✓** | **✓** |  | **✓** | **✓** |  | **✓** | **✓** | **✓** | **✓** |  |
| Behavioural practice/rehearsal |  | **✓** |  | **✓** | **✓** |  |  | **✓** | **✓** | **✓** | **✓** | **✓** |
| Credible source |  | **✓** | **✓** |  |  |  |  | **✓** | **✓** | **✓** | **✓** | **✓** |
| Feedback on behaviour | **✓** | **✓** | **✓** | **✓** |  |  | **✓** |  | **✓** | **✓** | **✓** | **✓** |
| Goal setting (behaviour) | **✓** | **✓** | **✓** |  | **✓** | **✓** | **✓** | **✓** | **✓** | **✓** | **✓** | **✓** |
| Goal setting (outcome) | **✓** | **✓** | **✓** |  | **✓** | **✓** | **✓** | **✓** | **✓** | **✓** | **✓** | **✓** |
| Graded tasks |  | **✓** | **✓** |  | **✓** | **✓** |  |  | **✓** |  | **✓** | **✓** |
| Information about health consequences |  | **✓** | **✓** |  | **✓** | **✓** |  | **✓** | **✓** | **✓** | **✓** | **✓** |
| Monitoring of outcome(s) of behaviour without feedback |  |  |  |  |  |  |  |  |  |  |  |  |
| Pharmacological support |  | **✓** |  |  |  |  |  |  |  |  |  |  |
| Problem solving | **✓** | **✓** | **✓** |  | **✓** | **✓** |  | **✓** | **✓** | **✓** | **✓** | **✓** |
| Pros and cons |  |  | **✓** |  |  |  |  | **✓** |  |  |  |  |
| Review outcome goal(s) |  | **✓** | **✓** |  | **✓** |  |  | **✓** |  |  | **✓** |  |
| Self-monitoring of behaviour | **✓** | **✓** | **✓** | **✓** | **✓** |  | **✓** |  | **✓** | **✓** | **✓** | **✓** |
| Self-monitoring of outcome(s) of behaviour | **✓** | **✓** | **✓** | **✓** | **✓** |  | **✓** | **✓** | **✓** | **✓** | **✓** | **✓** |
| Social support (emotional) |  |  |  |  | **✓** |  |  |  |  |  | **✓** |  |
| Social support (practical) | **✓** | **✓** |  |  | **✓** |  |  |  |  |  |  |  |
| Social support (unspecified) | **✓** | **✓** | **✓** | **✓** | **✓** | **✓** | **✓** | **✓** | **✓** | **✓** | **✓** | **✓** |
| Adding objects to the environment |  |  |  |  | **✓** |  |  | **✓** |  |  | **✓** |  |
| Anticipated regret |  | **✓** |  |  | **✓** |  |  |  |  |  | **✓** |  |
| Associative learning |  |  | **✓** |  | **✓** |  |  |  |  |  | **✓** |  |
| Avoidance/reducing exposure to cues for the behaviour |  | **✓** | **✓** |  | **✓** |  |  | **✓** |  |  | **✓** |  |
| Biofeedback |  | **✓** |  |  |  |  |  |  |  |  |  |  |
| Body changes |  | **✓** | **✓** |  |  |  |  |  |  |  | **✓** |  |
| Commitment |  | **✓** |  |  | **✓** |  | **✓** |  |  |  | **✓** |  |
| Comparative imaginings of future outcomes |  | **✓** | **✓** |  |  |  |  |  | **✓** |  |  |  |
| Demonstration of behaviour |  | **✓** |  | **✓** | **✓** |  |  |  |  | **✓** | **✓** |  |
| Discrepancy between current behaviour and goal |  |  |  |  |  |  |  |  |  | **✓** | **✓** |  |
| Distraction |  | **✓** |  |  |  |  |  |  |  | **✓** | **✓** |  |
| Feedback on outcome(s) of behaviour | **✓** | **✓** | **✓** | **✓** | **✓** |  | **✓** | **✓** |  | **✓** | **✓** |  |
| Focus on past success |  | **✓** | **✓** |  | **✓** |  |  | **✓** |  | **✓** | **✓** | **✓** |
| Framing/reframing |  | **✓** | **✓** |  | **✓** |  |  | **✓** |  | **✓** | **✓** | **✓** |
| Habit formation |  | **✓** |  |  | **✓** |  |  |  | **✓** |  | **✓** | **✓** |
| Habit reversal |  |  |  |  | **✓** |  |  | **✓** |  |  | **✓** |  |
| Identification of self as role model |  | **✓** |  |  |  |  |  |  |  |  |  |  |
| Identity associated with changed behaviour |  |  |  |  |  |  |  |  |  |  | **✓** |  |
| Imaginary reward |  |  |  |  | **✓** |  |  |  |  |  |  |  |
| Increase positive emotions |  | **✓** |  |  | **✓** |  |  | **✓** |  | **✓** | **✓** |  |
| Information about antecedents |  | **✓** | **✓** |  | **✓** |  |  | **✓** |  | **✓** | **✓** | **✓** |
| Information about emotional consequences |  | **✓** | **✓** |  | **✓** |  |  | **✓** |  | **✓** | **✓** |  |
| Information about social and environmental consequences |  | **✓** |  |  | **✓** |  |  | **✓** |  |  | **✓** |  |
| Instruction on how to perform the behaviour |  | **✓** |  | **✓** | **✓** |  |  | **✓** |  | **✓** | **✓** |  |
| Material incentive (behaviour) |  |  |  |  | **✓** |  |  |  |  |  |  |  |
| Material reward (behaviour) |  | **✓** |  |  | **✓** |  |  |  |  |  |  |  |
| Mental rehearsal of successful performance |  | **✓** |  |  | **✓** |  |  |  | **✓** |  | **✓** |  |
| Monitoring emotional consequences |  | **✓** | **✓** |  |  |  |  |  | **✓** |  | **✓** |  |
| Prompts/cues |  | **✓** | **✓** |  | **✓** | **✓** | **✓** | **✓** | **✓** | **✓** | **✓** |  |
| Re-attribution |  |  |  |  |  |  |  |  |  | **✓** | **✓** | **✓** |
| Reduce negative emotions |  | **✓** | **✓** | **✓** | **✓** |  |  | **✓** |  | **✓** | **✓** |  |
| Remove access to the reward |  | **✓** |  |  |  |  |  | **✓** |  |  | **✓** |  |
| Restructuring the physical environment |  | **✓** | **✓** |  | **✓** |  |  | **✓** | **✓** |  | **✓** |  |
| Restructuring the social environment |  |  | **✓** |  |  |  |  | **✓** | **✓** |  | **✓** |  |
| Review behaviour goal(s) | **✓** | **✓** | **✓** |  | **✓** |  |  | **✓** | **✓** |  | **✓** |  |
| Reward (outcome) |  | **✓** | **✓** |  | **✓** | **✓** |  |  | **✓** |  | **✓** |  |
| Salience of behaviours |  | **✓** |  |  |  |  |  | **✓** |  |  | **✓** |  |
| Salience of consequences |  |  |  |  |  |  |  |  |  |  | **✓** |  |
| Self-reward |  | **✓** |  |  | **✓** |  | **✓** |  |  |  | **✓** |  |
| Self-talk |  | **✓** |  |  | **✓** |  |  |  |  |  |  |  |
| Social comparison |  |  |  |  |  |  |  |  |  | **✓** | **✓** | **✓** |
| Social incentive |  |  |  | **✓** | **✓** |  |  |  |  |  |  |  |
| Social reward |  | **✓** | **✓** | **✓** | **✓** | **✓** |  |  | **✓** |  | **✓** | **✓** |
| Valued self-identity |  | **✓** |  |  | **✓** |  |  |  |  |  |  |  |
| Verbal persuasion about capability |  |  |  |  | **✓** |  |  |  |  |  |  |  |
| **Totals:** | **11/51** | **48/51** | **31/51** | **11/46** | **45/46** | **11/46** | **11/41** | **30/41** | **23/41** | **26/50** | **50/50** | **19/50** |
| **Percent:** | **22%** | **94%** | **61%** | **24%** | **98%** | **24%** | **27%** | **73%** | **56%** | **52%** | **100%** | **38%** |

***Note.* The BCTs with red ticks indicate those which were either only prompted in providers’ programmes, or delivered via external sources only, thus are included in the sensitivity analysis.**

*Note.* BCTs above the bold line are those 19 BCTs which were specified for inclusion in the NHS-DPP programme specification.

*^a^* We were not able to analyse the group support functionalities offered by all providers due to data protection considerations. Results may have differed if the research team could access these groups. This may impact results for Provider D who delivered health coaching via moderation of peer support groups.

*^b^* Note that provider only supplied research team with an app user guide, which outlined what was available via the app and how to use functions of the app, but researchers did not have direct access to the provider app. Thus, results may have varied if actual access to the app was provided.

*^c^* Data from interviews with health coaches about their job role as health coach on the NHS-DDPP, and includes data from audio-recorded telephone consultations between health coaches and service users for provider A.
